# Which instruments are used to measure shared, supported and assisted healthcare decision-making between patients who have limited, impaired or fluctuating capacity, their family carers and healthcare professionals? A systematic review protocol

**DOI:** 10.12688/hrbopenres.12932.2

**Published:** 2020-11-23

**Authors:** Francesco Fattori, Deirdre O'Donnell, Beatriz Rodríguez-Martín, Thilo Kroll

**Affiliations:** 1 School of Nursing, Midwifery and Health Systems, University College Dublin, Dublin, Ireland; 2 Faculty of Health Sciences, University of Castilla-La Mancha, Toledo, Spain

**Keywords:** Shared decision-making, Supported decision-making, Assisted decision-making, Capacity Mental competency, Personal autonomy, Patient participation, Physician-patient relations

## Abstract

**Background: **Shared decision-making (SDM) is a dialogical relationship where the physician and the patient define the problem, discuss the available options according to the patient’s values and preferences, and co-construct the treatment plan. Undertaking SDM in a clinical setting with patients who have limited, impaired or fluctuating cognitive capacity may prove challenging. Supported (defined “Assisted” in the Irish context) decision-making describes how people with impaired or fluctuating capacity remain in control of their healthcare-related choices through mechanisms which build and maximise capacity.

Supported and assisted decision-making (ADM) within healthcare settings is theoretically and practically novel. Therefore, there is a knowledge gap about the validity of psychometric instruments used to assess ADM and its components within clinical settings. This systematic review aims to identify and characterise instruments currently used to assess shared, supported and assisted healthcare decision-making between patients with limited, impaired or fluctuating capacity, their family carers and healthcare professionals.

**Methods: **A systematic review and narrative synthesis will be performed using a search strategy involving the following databases (PubMed, Cinahl, Embase, Web of Science, Scopus and PsycINFO). Quantitative studies published in the last decade and describing psychometric instruments measuring SDM, supported decision-making and ADM with people having limited or fluctuating capacity will be considered eligible for inclusion. Title and abstract screening will be followed by full-text eligibility screening, data extraction, synthesis and analysis. This review will be structured and reported according to the PRISMA checklist. The COSMIN Risk of bias checklist will be used to assess the quality of the instruments.

**Discussion: **The results will inform and be useful to HCPs and policymakers interested in having updated knowledge of the available instruments to assess SDM, supported and assisted healthcare decision-making between patients who have impaired or fluctuating capacity, their family carers and healthcare professionals.

**Registration: **PROSPERO **** CRD42018105360; registered on 10/08/2018.

## Introduction

Person-centred healthcare promotes the autonomy of persons about their treatment choices and places patients at the centre of care planning, considering them as partners in the decision-making process ( Kusnanto, 2018; Tullo *et al*., 2018). Considering patients' will and preferences in the development of care plans and decision-making related to medical treatment choices is central to person-centred healthcare ( Mulley *et al*., 2012). Research has highlighted that informed patients and families, receptive healthcare professionals, as well as coordinated and supportive healthcare environments, are crucial elements in the implementation of a patient-centred approach to health service planning and delivery ( Epstein *et al*., 2010). Patient participation in care planning and treatment decision-making is the result of a cultural shift in historically paternalistic healthcare settings ( Weston, 2001). This shift recognises the importance of considering patients’ will and preferences in the development of care plans and decision-making related to medical treatment choices ( Mulley *et al*., 2012). The research evidence has highlighted that informed patients and families, receptive healthcare professionals, as well as coordinated and supportive healthcare environments, are crucial elements in the implementation of a patient-centred approach to health service planning and delivery ( Epstein *et al*., 2010).

This drive towards developing a culture of person-centredness and patient participation in healthcare policy, research, and service delivery has instigated a growing body of literature which is exploring patient engagement in care planning ( Angel & Frederiksen, 2015). This literature focuses on the dynamic relationships between healthcare practitioners (HCPs), patients and their family caregivers as well as themes such as time, knowledge, the patient’s situation and HCPs attitudes (ibidem; Davies *et al*., personal communication). Consequently, several new concepts have arisen in the last decade that describe the different roles that patients may assume within the healthcare system: patient participation, patient activation, patient engagement, shared decision-making (SDM), supported decision-making and patient involvement among the others ( Barello *et al*., 2016; Browning *et al*., 2014; O’Donnell *et al*., 2018).

## Shared decision-making and its recent development

Shared decision-making (SDM) is characterised by a dialogical relationship where the physician and the patient define the problem, discuss the available options according to the patient’s values and preferences, and co-construct the treatment plan ( Makoul & Clayman, 2006). The dynamic nature and complexity of the concept of shared decision-making has challenged scholars seeking to define it ( Makoul & Clayman, 2006). SDM with patients who have limited, impaired or fluctuating cognitive capacity is defined supported decision-making (i.e. in Canada, Australia and US) or assisted decision-making (in Ireland) and may prove challenging. In this instance, specific support and assistance is required to build a patient’s capacity and to enable shared decision-making. Supported decision-making describes how people with impaired or fluctuating capacity remain in control of their healthcare-related choices through mechanisms which build and maximise capacity ( Browning *et al*., 2014; Davidson *et al*., 2015). These supportive mechanisms may include nominated decision-supporters, decision-making aids or assistive communication technologies.

Since 2006, successive states have ratified the United Nations Convention on the Rights of Persons with Disabilities (UNCRPD) (2006). This is a human rights instrument which aims to protect the autonomy and promote the full participation of people with disabilities within international human rights laws. The convention protects the rights of people with disabilities to autonomy and participation in all decisions which affect their lives, including healthcare decisions. Furthermore, it enshrines the right for people to have their decision-making capacity supported through an informed decision-making process (Davies *et al*., *in publication*).

Both supported and assisted decision-making are challenging constructs in terms of definition and implementation ( Browning *et al*., 2014; O’Donnell *et al*., 2018). They can both be considered and defined as a process, a legal framework, a mechanism and a system, making its implementation and translation into practice harder ( Browning *et al*., 2014; Kohn & Blumenthal, 2014). Assisted and Supported decision-making describe the process leading people with impaired or fluctuating capacity to remain in control of their healthcare-related choices through mechanisms which build and maximise capacity. These supportive mechanisms may include nominated decision-supporters, decision-making aids or assistive communication technologies (insert citation).

Several different instruments have been developed to assess the complex and multifaceted phenomenon of shared decision-making in the healthcare setting ( Bouniols *et al*., 2016; Makoul & Clayman, 2006; Scholl *et al*., 2011; Simon *et al*., 2007). These scales measure different components or phases in the SDM process between physicians and patients having cognitive capacity, such as antecedents of the decision-making process, the process itself and the decision outcomes ( Perestelo-Perez *et al*., 2017; Scholl *et al*., 2011).

Several systematic reviews have been undertaken within the last 20 years, which have mainly focused on the retrieval and analysis of instruments assessing the different components of SDM within healthcare settings between patients with mental capacity and physicians. Elwyn *et al*. (2001) could not retrieve any study evaluating the involvement of patients with assumed or established limited cognitive capacity in shared decision-making. Six years later, Simon *et al*. (2007) were able to identify 18 instruments which measured the patients’ perspective, preferences for information and participation, decisional conflict, self-efficacy as well as the evaluation of the decision-making process and outcomes. Scholl *et al*. (2011) contributed to this growing area retrieving 28 scales, underlining their development and validation in languages other than English, recognising an increasing internationalisation of SDM. Recently, 19 studies have been included by Bouniols *et al*. (2016), highlighting the evolution of instruments which take into account points of view of patients, HCPs and external observers. Recently, Perestelo-Perez *et al.* (2017) focused on SDM measures within the mental health area and reported 48 instruments, mainly assessing the SDM process from the patients’ perspective.

Because of the novelty of concepts such as supported and assisted decision-making, there is no reference within the scientific literature about instruments able to assess these processes in the clinical practice. Accordingly, for this review, we consider shared decision-making instruments used with cohorts of people with a physical, age-related or mental health condition that may lead to limited, impaired and fluctuating capacity as plausible measures of supported and assisted decision-making.

This review answers the call for further research about decision-making processes among cohorts of people with potential impaired or limited capacity ( Duncan *et al*., 2010) and it represents an extension of the recent works by Bouniols *et al*. (2016); Scholl *et al*. (2011); Simon *et al*. (2007) and Perestelo-Perez *et al*. (2017)..

This systematic review aims to identify and synthesise the instruments used to measure shared, supported and assisted healthcare decision-making between patients who have limited, impaired or fluctuating capacity, their family carers and healthcare professionals.

## Review question

Which instruments are used to assess shared, supported and assisted healthcare decision-making between patients who have limited, impaired or fluctuating capacity, their family carers and healthcare professionals?

## Method

### Selection and inclusion criteria

The following selection inclusion criteria will be considered:

a) Peer-reviewed quantitative scientific studies;
b) Materials are written in the English language;
c) 10-year time frame (2009–2019);
d) Human subjects (+18 years old) as participants;
e) Papers describing psychometric instruments as objects of a creation and validation study or used as part of a battery within a broader study;
f) Instruments assessing SDM, supported or assisted decision-making related antecedents, process and outcomes constructs;
g) The population targeted by the instruments will include: People presenting limited, impaired or fluctuating capacity due to a physical or diagnosed mental health condition; healthcare professionals of any type (i.e. physicians, nurses, occupational therapist, physiotherapist and so on) working in primary, secondary and tertiary care such as hospitals, nursing homes, psychiatric hospitals and rehabilitation hospitals; family members and patient nominees acting as surrogate or decision-making supporters;
h) The outcomes will be direct patient-reported outcome measures or family carer-reported outcome measures, clinician-reported outcome measures and objective observer-based outcome measures.

### Exclusion criteria

a) Studies, where the instruments are used with mixed samples of people with and without capacity impairments and the results are not disaggregated;
b) Dissertations or theses;
c) We will exclude those instruments that the authors do not explicitly consider as measures of SDM, supported or assisted decision-making even if labelled otherwise (e.g. decisional conflict scale not used as a measure of SDM).

### Information sources

Two authors developed and agreed on the search strategy (keywords, subject headings, limiters, and so on). Two authors (FF, BRM) will run the search independently on the following electronic databases Cinahl, PubMed, Embase, Web of Science, Scopus and PsycINFO. The results of the independent searches will be exported and uploaded on a reference management software (Zotero). After the elimination of the duplicates, and as highlighted previously, the above-cited limits will apply to the search (please refer to paragraph *Selection criteria*). The first search will be run in July 2019. As a secondary search, reference lists of the included full-texts will be used as a further retrieval source.

### Search strategy

The keywords composing the search strings have been adapted from a previous realist review of research evidence concerning the mechanisms which support ADM in healthcare settings ( Davies *et al*., 2019). This previous search strategy was modified according to the aims of the present review and will be used as the basis for the development of the database-specific (Mesh and Headings) search strategies as outlined here below in Table 1.

**Table 1.** Search strategy, keywords and Boolean operators.

assisted decision-making OR supported decision-making OR shared decision-making OR patient participation OR patient activation OR patient engagement OR patient decision-making OR physician-patient relation OR doctor-patient relation OR nurse-patient relation OR social worker-patient relation OR patient-family relation OR professional-patient disagreement OR professional-family relations OR advance care planning
AND
intervention OR education OR training OR program OR activity OR initiative OR efficacy
AND
cognitive impairment OR intellectual disability OR dementia OR brain injury OR dysphasia OR communication skills OR communication disorders OR mental disorder OR neurocognitive disorder OR decision competency OR decision capacity OR vulnerable populations OR mental competency OR information literacy
AND
randomised controlled trial OR controlled clinical trial OR randomised OR placebo OR randomly OR trial OR groups OR nonrandomised controlled trial OR cohort OR control OR validation OR longitudinal OR observational

We will use a flow diagram to report the inclusion and eligibility process and a table to describe the main features of the studies included in the final review. The full search strategies for the three databases with relative Boolean rationales will be reported in the appendix.

### Studies selection and screening criteria

Initially, the authors will retrieve the initial pool of studies from the databases following the inclusion criteria described above. We will use Zotero to remove duplicates. Then, we will proceed with the title and abstract screening. FF and BRM will conduct the screening and the full-texts review phase independently and will identify cases which require discussion and resolution by consensus. DOD will verify a sub-proportion of the texts, and the inter-rater agreement will be assessed. Again, disagreements will be solved by a discussion between FF and BRM; if a solution cannot be found, DOD will decide.

### Data extraction

FF and DOD will develop the data extraction and synthesis form inspired by Makoul and Clayman’s integrative model (2006) (nine essential elements), adding the description of the type of mental health condition ( Perestelo-Perez *et al*., 2017). The extraction table will include the: name of the tool, measured variable, the tool’s version, complete reference, study design, population, study methods, point of view, mental condition, number of dimensions and items, response scale, reliability and validity indexes.

### Assessment of the quality of the studies and instruments

FF and BRM will proceed to the assessment of the quality of both the instruments and the papers independently. Any disagreement will be referred to a third reviewer (DOD). The quality of the retrieved instruments will be assessed with the COSMIN Risk of bias checklist ( Mokkink *et al*., 2018). This appraisal will evaluate the methodological rigour of the validation process and the psychometric properties of the instruments. The instruments will be presented in such a way as to align them with patient characteristics, healthcare setting and outcome.

### Data synthesis

Findings will be synthesised using a narrative synthesis approach ( Popay *et al*., 2006). There is currently no method to empirically group measurement properties; synthesis is then recommended ( Beattie *et al*., 2014). We will categorise the instruments according to the assessed variable, their format, and what feature of the ADM process investigate (antecedents, the process itself or the outcomes).

We will also discuss patterns of recurrences and differences between the instruments, uncovering and highlighting the communal components, identifying patterns that lead to the utilisation of effective tools assessing ADM with people with limited, impaired or fluctuating capacity.

### Study registration

The systematic review is registered with the protocol number CRD42018105360 (10/08/2018) in the PROSPERO register.

## Discussion

The systematic review aims to analyse the last ten years of development of psychometric instrument assessing shared decision-making with people with limited, impaired or fluctuating capacity, supported and assisted decision-making. Due to the novelty of supported and assisted decision-making constructs, we explicitly chose to address instruments assessing shared decision-making with people who have limited, impaired or fluctuating capacity. This will allow us to formulate plausible inferences about the validity of tools which measure supported and assisted decision-making.

By considering instruments assessing supported decision-making processes with samples of people with limited, impaired and fluctuating capacity, this review will inform practice development about building and supporting decision-making with this cohort of patients. Through the categorisation of the results, policy-makers and managers of healthcare settings may find useful insights on which instruments are more appropriate than others to assess different components of the complex process of supporting the decision-making of patients who have limited, impaired or fluctuating capacity.

## Dissemination of the review

The results of this review will be published in peer-reviewed journals (e.g. Health Expectations) and presented during academic conferences. We will also share the findings in accessible and appropriate formats (e.g. short infographics) with non-academic readers.

## Data availability

### Underlying data

No underlying data are associated with this article.

### Reporting guidelines

Figshare: PRISMA-P checklist for “Which instruments are used to measure shared, supported and assisted healthcare decision-making between patients who have limited, impaired or fluctuating capacity, their family carers and healthcare professionals? A Systematic Review Protocol”. https://doi.org/10.6084/m9.figshare.9366347.v1 ( Fattori *et al*., 2019).

The completed checklist is available under the terms of the Creative Commons Attribution 4.0 International license (CC-BY 4.0).
