# Supplementary material for: Which instruments are used to measure shared, supported and assisted healthcare decision-making between patients who have limited, impaired or fluctuating capacity, their family carers and healthcare professionals? A systematic review protocol
Source: HRB Open Res. 2020 Nov 23;2:19. Originally published 2019 Aug 16. [Version 2] doi: 10.12688/hrbopenres.12932.2 (PMC8039860; doi:10.12688/hrbopenres.12932.2)
Supplement: Peer review history

# Peer review: Reviewer response for version 2

**DOI:** 10.21956/hrbopenres.14317.r29183

**Published:** 2021-04-09

**Authors:** Paul Webb

**Affiliations:** 1 Praxis Care, Belfast, UK

Thank you for inviting me to review a study protocol for a systematic review and narrative synthesis of the psychometric instruments which are used to measure shared, supported and assisted healthcare decision-making between patients who may have limited and impaired capacities or capacities which change through time and their family carers and healthcare professionals.

 I think that this study could potentially be an important contribution to the literature as well as being an excellent resource for healthcare practitioners (HCPs). In general, the study protocol is well written although I’m not entirely sure that key constructs like SDM and ADM are described in sufficient detail and that the study design is wholly appropriate to the research question. My reasoning is as follows:

1. Shared decision-making, supported decision-making and assisted decision-making are not clearly defined in the study protocol e.g. “SDM with patients who have limited, impaired or fluctuating cognitive capacity is defined supported decision-making (i.e. in Canada, Australia and US) or assisted decision-making (Ireland) and may prove challenging” (p. 3). A bit more clarity and detail may perhaps be needed so that the reader can understand the distinctions between shared decision-making, supported decision-making and assisted decision-making and thereby appreciate what the respective instruments are trying to measure.
2. It’s unclear from the study protocol whether the scope of the systematic review and narrative synthesis includes psychometric instruments which measure SDM, supported decision-making and ADM or whether the scope is, in effect, restricted to instruments which measure SDM alone. The authors state that “this systematic review aims to identify and characterize instruments currently used to assess shared, supported and assisted healthcare decision-making” (p. 1) whilst writing further on in the study protocol that “there is no reference within the scientific literature” (p. 4) to instruments which can assess supported decision-making and ADM.
3. The authors then suggest that shared decision-making instruments can be “plausible measures of supported and assisted decision-making” (p. 4) which seems to confirm that there are a lack of instruments assessing supported decision-making and ADM. In the absence of instruments which assess supported decision-making and ADM, what’s the rationale for assuming that shared decision-making instruments can be used as measures of supported and assisted decision-making? If there isn’t a convincing rationale, then the scope of the systematic review may be narrower than the study protocol suggests.
4. Moving on to the selection inclusion criteria, the target population on page 4 includes health professionals i.e. ‘physician’, ‘nurse’, ‘occupational therapist’ and ‘physiotherapist’. However, ‘occupational therapist’ and ‘physiotherapist’ are not included in the search terms listed in Table 1 (p. 5). In a similar vein, ‘psychologist’ is omitted from the list of search terms too. Is there a rationale for this? Conversely, ‘social worker’ is not listed as an example of a healthcare professional in the selection inclusion criteria but appears as a search term in Table 1. Is there a rationale for this?

I hope that this review is of assistance and thank you again for the invitation to review this study protocol.

Is the study design appropriate for the research question?

Partly

Is the rationale for, and objectives of, the study clearly described?

Partly

Are sufficient details of the methods provided to allow replication by others?

Yes

Are the datasets clearly presented in a useable and accessible format?

Not applicable

Reviewer Expertise:

Supported decision-making, co-production, physical activity.

I confirm that I have read this submission and believe that I have an appropriate level of expertise to confirm that it is of an acceptable scientific standard, however I have significant reservations, as outlined above.

# Peer review: Reviewer response for version 2

**DOI:** 10.21956/hrbopenres.14317.r28417

**Published:** 2020-12-03

**Authors:** Craig Sinclair

**Affiliations:** 1 Centre of Excellence in Population Ageing Research, University of New South Wales, Sydney, Australia

The authors have answered my main points from the initial review. I noticed that there is a missing citation in paragraph 5.

Is the study design appropriate for the research question?

Partly

Is the rationale for, and objectives of, the study clearly described?

Yes

Are sufficient details of the methods provided to allow replication by others?

Yes

Are the datasets clearly presented in a useable and accessible format?

Not applicable

Reviewer Expertise:

Psychology, health services research, older adult decision-making, dementia, supported decision-making, advance care planning.

I confirm that I have read this submission and believe that I have an appropriate level of expertise to confirm that it is of an acceptable scientific standard.

# Peer review: Reviewer response for version 1

**DOI:** 10.21956/hrbopenres.14012.r27542

**Published:** 2020-06-29

**Authors:** Craig Sinclair

**Affiliations:** 1 Centre of Excellence in Population Ageing Research, University of New South Wales, Sydney, Australia

Thank you for the opportunity to review this paper, which describes the protocol for a systematic review and narrative synthesis of peer-reviewed studies of psychometric instruments measuring shared decision-making, supported decision-making and assisted decision-making among people with limited or fluctuating decision-making capacity. This important study addresses the need for an up-to-date review of the tools used to assess decision-making involvement in healthcare decision-making, among those with limited or fluctuating decision-making capacity.

 I found the protocol to be clearly written, and overall to describe a suitably rigorous approach to the review. I did have two reservations, which are described below. These relate to the clarity of the protocol description and a potential issue with the exclusion of relevant papers from the observational research literature.

 Point 1: On p3 the authors write "Accordingly, for this review, we consider shared and assisted decision-making instruments used with cohorts of people with a physical or mental health condition that may lead to limited, impaired and fluctuating capacity as plausible measures of supported and assisted decision-making." While it becomes clear later in the manuscript that tools relevant for participants with age-related cognitive impairments are included in the review, this definition is potentially confusing in its implication that measures/instruments used among people with age-related cognitive impairments (which are not necessarily a mental health condition) are not included. This could be addressed relatively easily.

 Point 2: On p4, the final search term string is defined as "randomised controlled trial OR controlled clinical trial OR randomised OR placebo OR randomly OR trial OR groups OR nonrandomised controlled trial OR cohort OR control". I am surprised that the terms here are so narrow, potentially excluding psychometric validation studies, epidemiological and/or observational studies, all of which might contribute useful tools for measurement of shared, supported or assisted decision-making. I would recommend the inclusion of terms "validation", "longitudinal" and "observational".

 Thank you once again for the opportunity to contribute to this work.

Is the study design appropriate for the research question?

Partly

Is the rationale for, and objectives of, the study clearly described?

Yes

Are sufficient details of the methods provided to allow replication by others?

Yes

Are the datasets clearly presented in a useable and accessible format?

Not applicable

Reviewer Expertise:

Psychology, health services research, older adult decision-making, dementia, supported decision-making, advance care planning.

I confirm that I have read this submission and believe that I have an appropriate level of expertise to confirm that it is of an acceptable scientific standard, however I have significant reservations, as outlined above.

# Peer review: Reviewer response for version 1

**DOI:** 10.21956/hrbopenres.14012.r27351

**Published:** 2020-05-26

**Authors:** Lee A. Fleisher, Justin Clapp

**Affiliations:** 1 Department of Anesthesiology and Critical Care, University of Pennsylvania, Philadelphia, PA, USA

This study protocol for a systematic review of instruments designed to measure decision making in cognitively impaired patients is by and large methodologically sound. It could mainly use improvement in explaining its rationale and research question.

 As a central premise motivating the intended study, the authors state that patient participation in decision making improves clinical outcomes, quality of care, and patient safety. However, they cite only one article to support this substantial claim (Schwappach *et al.*), and they overstate the findings of this article, which is a review of patient involvement in error prevention—not shared decision making. The authors should more substantially assess this premise through a more thorough exploration of the literature on shared decision making (for example, the Cochrane review by Stacey *et al.*). Interventions such as decision aids intended to support shared decision making have shown mixed findings as to their ability to improve care—one major reason why further study is necessary.

 This is slippage in the rationale between the concepts of shared decision making, supported decision making, and assisted decision making (which leads to confusion in the methods, since these are the terms guiding the review). What’s the difference between supported and assisted decision making? The terms all need to be clearly defined and related to one another. Similarly, in the paragraph where the authors summarize previous reviews on shared decision-making instruments, it is not clear if these reviews included instruments intended to measure supported/assisted decision making.

 Finally, the precise gap being addressed by the research question is not quite clear. Is the problem that there are no instruments (as the introductory sections seem to indicate) measuring supported/assisted decision making or that there has been no assessment of these instruments?  If there are very few instruments, won’t the review have very little to demonstrate (as it will just serve as another review of shared decision-making instruments)?

Is the study design appropriate for the research question?

Partly

Is the rationale for, and objectives of, the study clearly described?

Partly

Are sufficient details of the methods provided to allow replication by others?

Yes

Are the datasets clearly presented in a useable and accessible format?

Partly

Reviewer Expertise:

Shared decision making

We confirm that we have read this submission and believe that we have an appropriate level of expertise to confirm that it is of an acceptable scientific standard, however we have significant reservations, as outlined above.
